# Self-Management Behaviors in Obese Patients Undergoing Surgery Based on General and Specific Adherence Scales

**DOI:** 10.29252/wjps.8.1.85

**Published:** 2019-01

**Authors:** Zahra Sobhani, Masood Amini, Maryam Zarnaghash, Seyed Vahid Hosseini, Hamid Reza Foroutan

**Affiliations:** 1 Laparoscopy research center, Shiraz University of Medical Sciences, Shiraz, Iran;; 2 Department of Surgery, School of Medicine, Laparoscopy Research Center, Shiraz University of Medical Sciences, Shiraz, Iran;; 3 Department of Psychology and Educational Sciences, Marvdasht Branch, Islamic Azad University, Marvdasht, Iran;; 4 Department of Surgery, School of Medicine, Colorectal Research Center, Shiraz University of Medical Sciences, Shiraz, Iran

**Keywords:** General adherence, Specific adherence, Self-management behaviors, Surgery, Obesity

## Abstract

**BACKGROUND:**

Adherence has been defined as the degree to which a patient’s voluntary behavior corresponds with the clinical recommendations of health care providers. The aim of this study was to predict self-management behaviors in obese patients undergoing surgery based on general and specific adherence scales.

**METHODS:**

All obese patients who underwent bariatric surgery in Ghadir Mother and Child Hospital, Shiraz, Iran from April 2017 to September 2017 were enrolled. By using available sampling method, 201 patients with BMI above 35 (36.43±35.11) and in the age range of18-65 years (13.38±80/5) were selected. A questionnaire containing general adherence scale (GAS), specific adherence scale (SAS) and post-surgery self-management behaviors questionnaire (BSSQ) was completed by all patients.

**RESULTS:**

The general and specific adherence scales were significant predictors for self-management behaviors after surgery, and positive relation was found for general and specific adherence scales with self-management behaviors.

**CONCLUSION:**

Self-management behaviors regarding eating behaviors, supplements, fruits, vegetables, grains, protein and fluid intake, physical activity, dumping syndrome management have significant relationship with general and specific adherence scales. So increasing knowledge, skills, motivation, self-confidence, self-efficacy and self-monitoring of obese patients after surgery seem necessary.

## INTRODUCTION

Obesity is a public health problem that has raised concern worldwide and according to the World Health Organization (WHO), more than 1.9 billion people suffer from overweight and 600 million from obesity.^1^ Obesity can be defined as a condition of abnormal or excess fat accumulation in adipose tissue, to the extent that health may be impaired.^2^ Overweight and obesity are major causes of co-morbidities, including type II diabetes, cardiovascular diseases, various cancers and other health problems, which can lead to further morbidity and mortality.^3^^,^^4^ Body Mass Index (BMI), which is calculated as [(weight in kg)/(height in m^2^)], is considered to be the most useful population-level measure of obesity, and it is a simple index to classify underweight, overweight and obesity in adults. The WHO has classified overweight and obesity in adults based on various BMI cutoffs.^2^

Since the 1970’s, there has been a 300% increase in the prevalence of adolescent extreme obesity.^5^^-^^7^ This shocking statistic coupled with the lack of effective behavioral and pharmacological weight loss treatments and the significant medical and psychological comorbidities of extreme obesity, has led to the acceptance of aggressive weight management behaviors, such as liposuction and bariatric surgery.^8^^-^^10^ Bariatric surgery is an effective treatment for patients with severe obesity as it leads to significant and sustainable weight loss and improvement in obesity-related comorbid conditions.^11^

During the last 10 years, there has been a significant increase in the prevalence of obesity, and in particular of morbid obesity,^12^ and the number of bariatric operations have more than doubled.^13^ There are three main kinds of surgical techniques, malabsortive and restrictive (with the former being considered more effective than the latter as to weight loss) and gastric bypass, which is considered a mixed approach.^14^ Weight loss after bariatric surgery is associated with regular attendance to postoperative follow-up.^15^^,^^16^ Successful weight loss is defined as more than 50% excess weight loss (EWL). In a review of the clinical effectiveness of bariatric surgery, percent EWL was reported to be between 63% and 78% following gastric bypass surgery.^11^ Yet, in a meta-analysis of the effectiveness of surgical treatment of obesity, 15–20% of the patients failed to achieve 50% EWL.^17^

Adherence has been defined as the degree to which a patient’s voluntary behavior corresponds with the clinical recommendations of health care providers^18^ and suggests that patients are self-sufficient individuals who assume an active and voluntary role in defining and achieving goals for their medical treatment.^18^ Adherence is a multi-dimensional element of the physical, mental, emotional and social status of patients.^19^ Adherence is very essential in chronic disease, like end-stage renal disease and maintenance dialysis,^20^^,^^21^ chronic heart failure,^22^ hypertension,^23^ type 2 diabetes,^24^ rheumatoid arthritis, breast cancer and obesity.^19^^,^^25^

This growing interest on the part of the scholars towards adherence issues has also characterized the scientific community dealing with the assessment of adherence in patients undergoing bariatric surgery and little research has examined the role of post bariatric surgery self-management behaviors adherence in predicting weight loss outcomes. Although bariatric surgery leads to successful weight loss for most patients, longitudinal work suggests a substantial minority (up to 30%) fail to lose weight, or regain a significant amount of weight over time.^26^ Bariatric surgery requires multiple postoperative lifestyle changes in diet, eating behavior, physical activity, and supplements consumption.^27^^,^^28^ Changing the lifestyle and healthy eating habits are associated with weight loss and postoperative sustained improvement in quality of life.^29^^-^^32^

Bariatric surgery procedures are not expected to have unhealthy lifestyle habits that might reemerge following surgery, such as binge eating, grazing, emotional eating, night eating, drinking of high-calorie beverages, or return to a sedentary lifestyle.^14^^,^^33^ Adherence to the recommended life-style after surgery varies among patients, so the aim of this study was to predict of self-management behaviors adherence in patients undergoing bariatric surgery based on general adherence and specific adherence scales. 

## MATERIALS AND METHODS

The population included all obese patients who underwent bariatric surgery in Ghadir Mother and Child Hospital, Shiraz, southern Iran from April 2017 to September 2017. By using available sampling method, 201 patients with at least one month and a maximum of three months after the surgery, with a BMI above 35 (36.43±35.11) and in the age range of 18-65 years (13.38±80.5), were selected. Of these sample, 149 (66%) were female and 52 (24%) were male, 69 (30.1%) were single and 132 (4.58%) were married. Exclusion criteria included catching learning disability, psychiatric treatment, or have serious medical problems such as chronic diseases other than obesity. The stages of this research implementation were approved by the Ethics Committee of Shiraz University of Medical Sciences (no. 1396.S404).

After explaining the importance and aim of research to the participants, with an emphasis on confidentiality; Informed consent to participate in research, and demographic profile questionnaire (age, sex, economic, social, marital, physical and mental health status, and surgical date) were provided and completed. Post-bariatric surgical self-management behaviors questionnaire (BSSQ), General Adherence Scale (GAS), Specific Adherence Scale (SAS) and necessary explanations for respond to questions, were given to participants. After completing the questionnaires by participants, they were evaluated by the researcher to ensure that patients have responded to all items.

The bariatric surgery self-management behaviors questionnaire (BSSQ) were developed by Welch *et al.*^34^ to assess self-management behaviors carried out over the previous week in brief, practical, and behavioral terms that would be acceptable to patients. BSSQ have 33 items with seven behavioral domains as (1) eating behaviors (eight EB items); (2) fluid intake (eight FI items); (3) protein intake (three PI items); (4) physical activity (three PA items); (5) dumping syndrome management (four DSM items); (6) fruit, vegetable, and whole grains intake (three FVW items); and (7) vitamin and mineral supplement intake (four SI items). 

High adherence to these behaviors was expected to enhance the likelihood of patient success after surgery in terms of excess weight loss and adequate nutritional status. BSSQ items have a Likert scale format of “never,” “sometimes,” or “always” and subscale and total scores converted to a 0–99 range for ease of interpretation, with higher scores indicating higher adherence. Psychometric analysis results of the seven BSSQ subscales and total test for internal reliability (coefficient alpha, α) were EB, α=0.83; FI, α=0.81; PI, α=0.74; PA, α=0.70; DSM, α=0.79; FVW, α=0.63; SI, α=0.79; and total score, α=0.83. Psychometric results for the LDQ were α=0.92, and those for the PBQ were α=0.78. Two-week test–retest reliabilities (ICC) for the BSSQ subscales were EB, ICC=0.72; FI, ICC=0.68; PI, ICC=0.60; PA, ICC=0.54; DSM, ICC=0.66; FVW, ICC=0.46; SI, ICC=0.66; and total score, ICC=0.71. 

Test–retest results for the LDQ were ICC=0.86, and those for the PBQ were ICC=0.64. BSSQ construct validity was examined by the pattern of inter correlations among individual BSSQ subscales, 19 of 21 correlations were significant (r=0.15, *p*<0.05 to r=0.39, *p*<0.01). Results of the correlations among the BSSQ total test and the LDQ, PBQ, and WRSM questionnaires and WL (weight loss) showed the total test correlated significantly with LDQ (r=−0.22, *p*<0.01), PBQ (r=0.31, *p*<0.01), and WRSM (r=−0.17, *p*<0.05), but not WL (r=−0.08, ns). 

Results of the BSSQ subscale correlations with the LDQ distress measure showed that EB, DSM, and PI subscales were significantly correlated with LDQ (r=−0.22 to r=−0.26, all *p*<0.01) but FI, PA, SI, and FVW were not. BSSQ subscales EB (r=0.33, *p*<0.01) and PA (r=0.23, *p*<0.01) correlated significantly with PBQ benefits but not FI, DSM, SI, FVW, or PI.^34^ In Sobhani *et al.*’s study,^35^ by using factor analysis method, 6 subscales for BSSQ were obtained in Iranian population (eating behaviors, fluid intake, vitamin and mineral supplement intake, fruit, vegetable, whole grains and protein intake, physical activity and dumping syndrome management), the specific values of each of the six subscales were 61.54% of the variance of self-management behaviors after bariatric surgery. The reliability coefficient for this questionnaire (Cronbach’s alpha=0.90) was obtained and using the split-correlation method, the two-part correlation was 0.78, indicating the desired reliability of the self-management behavior questionnaire after bariatric surgery.^35^

General and specific adherence scales, to evaluate adherence for patients with chronic disease in the medical outcome by Hayes *et al.*^36^ have been designed. General adherence scale measure the willingness of the patient to follow medical advice generally. This scale has five items and acceptable internal consistency (α=0.81) have been reported. Specific adherence scale measures to comply with the following recommendations for a disease. Specific adherence scale for cardiac patients measure cardiac patients to follow the recommendations of medication and lifestyle. The scale has 10 items in a Likert scale of six degrees of internal consistency obtained within acceptable limits (α=0.73). 

The reliability of this scale by Hayes and colleagues36 based on the correlation of the test-retest interval of two years is acceptable (SAS=0.55 and GAS=0.60). In this study, especially in patients with obesity dedicated following scale based on the following scale was adjusted for each patient heart that measures adherence of patients suffering from obesity advice on diet, supplements and lifestyle and alpha coefficients obtained for general adherence scale (0.76) and dedicated specific adherence scale (0.87) were obtained.

## Results

For considering role of predicting general adherence and specific adherence based on self-management behaviors adherence in patients undergoing bariatric surgery correlation and regression method were used. Table 1 shows the results of self-management behaviors between different subscales of BSSQ as well as a significant positive relationship between the agent and the total score. The concurrent validity of the implementation of self-management behavior questionnaire after surgery and follow up of the general and specific compliance were shown in Table 2. As can be seen in Table 3 and 4, general and specific adherence scales were significant predictors for self-management behaviors after surgery (*p*<0.0001 and *p*<0.0001). The results showed that general and specific adherence scales were significant predictors for self-management behaviors in patients undergoing bariatric surgery.

**Table 1:** The correlation matrix between factors with the total scores of self-management behaviors after surgery

**Variable**	**Eating behavior**	**Supplements**	**Physical activity**	**Vitamin, mineral, fruit, vegetable, whole grains and protein intake**	**Fluid intake**	**Dumping syndrome management**
Eating behaviors	1					
Supplements	0.53	1				
Physical activity	0.56	0.45	1			
vitamin and mineral supplement intake, fruit, vegetable, whole grains and protein intake	0.19	0.18	0.28	1		
Fluid intake	0.55	0.26	0.41	0.20	1	
Dumping syndrome management	0.52	0.33	0.29	0.12	0.45	1
Total	0.93	0.62	0.74	0.46	0.68	0.64

**Table 2:** The total score of the correlation matrix and self-management behaviors after surgery comply with the general and specific adherence scales

**Scale**	**Eating behavior**	**Supplements**	**Physical activity**	**Vitamin, minera, fruit, vegetable, whole grains and protein intake**	**Fluid intake**	**Dumping syndrome management**	**Total**
General adherence	0.36	0.09	0.17	0.25	0.24	0.28	0.36
Specific adherence scale	0.66	0.37	0.57	0.23	0.31	0.55	0.70

**Table 3:** Regression of self-management behaviors after surgery based on general and specific adherence scales

**Scale**	**R**	**R²**	**df**	**Adjusted** **R²**	**F**
General adherence	0.36	0.13	145	0.13	22.76
Specific adherence	0.70	0.49	144	0.49	140.2

**Table 4:** Regression of self-management behaviors after surgery based on specific adherence scales

**Scale**	**β**	**T**	**SD**	**B**	**P**
General adherence	0.36	4.77	0.15	0.75	0.0001
Specific adherence	0.70	11.84	0.06	0.73	0.0001

## DISCUSSION

The aim of this study was attempted to predict self-management behaviors in obese patients undergoing surgery based on general and specific adherence scales.The results showed that general and specific adherence scales were significant predictors for self-management behaviors in patients undergoing bariatric surgery. In the study of Salehi *et al.*, there was a significant reduction in weight in overweight patients between dialysis treatment sessions after receipt of an educational package.^37^ Waheedi *et al.* showed low adherence to self-care management and poor overall knowledge of patients that can be considred a big challenge in diabetes care in Kuwait.^38^

Some Studies show that assessment of psychological factors such as body image satisfaction, psychological well-being and quality of life are necessary in patients with bariatric surgery.^39^^,^^40^ In this study, self-management behaviors and follow-up specific and general treatments after bariatric surgery were evaluated; therefore, suffering from bariatric surgery may affect the perception of the disease, the level of self-control of self-management behaviors such as eating behavior, supplement, fruits, vegetables, grains and protein, and fluid intake, physical activity, management of dumping syndrome and general and specific adherence scales. The inefficiencies of self-management behaviors were found as side effects of medications, lack of adequate motivation and patient believes, inadequate counseling, and lack of supportive care.^41^

The adherence to therapeutic recommendations is a subject that has long been studied among patients and clinical professionals and was shown that failure to treatment can be an important health problem. Self-management behaviors have been studied in rheumatoid,^42^ heart disease,^43^ and diabetic patients,^44^ but there has been no research for patients undergoing bariatric surgery. Implementation of therapeutic educational plans in clinical practice has been undertaken and comprehensive self-management behaviors have been used to improve outcomes for patients with cardiovascular diseases.^45^ The results of our study showed that self-management behaviors regarding eating behaviors, supplements, fruits, vegetables, grains, protein and fluid intake, physical activity, dumping syndrome management have significant relationship with general and specific adherence scales. So increasing knowledge, skills, motivation, self-confidence, self-efficacy and self-monitoring of patients after the operation of obesity seem necessary.
